# The COMT-polymorphism is not associated with the incidence of acute kidney injury after cardiac surgery – a prospective cohort study

**DOI:** 10.1186/s12882-018-0820-x

**Published:** 2018-02-09

**Authors:** Mehmet Oezkur, Attila Magyar, Phillip Thomas, Andreas Reif, Stefan Störk, Peter U. Heuschmann, Rainer G. Leyh, Martin Wagner

**Affiliations:** 1 Department of Cardiovascular Surgery, University Hospital Würzburg, Würzburg, Germany; 2 Institute of Clinical Epidemiology and Biometry, University of Würzburg, Würzburg, Germany; 3 Clinical Trial Center Würzburg, University Hospital Würzburg, Würzburg, Germany; 4 Comprehensive Heart Failure Center, University of Würzburg, Würzburg, Germany; 5 Department of Medicine I, Division of Cardiology, University Hospital Würzburg, Würzburg, Germany; 6 Department of Medicine I, Division of Nephrology, University Hospital Würzburg, Würzburg, Germany; 7 Center of Mental Health, Department of Psychiatry, Psychosomatics and Psychotherapy, University Hospital Würzburg, Würzburg, Germany; 8 Department of Psychiatry and Psychotherapy, University Hospital Frankfurt, Frankfurt am Main, Germany

**Keywords:** AKI, KDIGO, Cardiac surgery, COMT

## Abstract

**Background:**

The Catechol-O-methyltransferase (COMT) represents the key enzyme in catecholamine degradation. Recent studies suggest that the COMT rs4680 polymorphism is associated with the response to endogenous and exogenous catecholamines. There are, however, conflicting data regarding the COMT Met/Met phenotype being associated with an increased risk of acute kidney injury (AKI) after cardiac surgery. The aim of the current study is to prospectively investigate the impact of the COMT rs4680 polymorphism on the incidence of AKI in patients undergoing cardiac surgery.

**Methods:**

In this prospective single center cohort study consecutive patients hospitalized for elective cardiac surgery including cardiopulmonary-bypass (CPB) were screened for participation. Demographic clinical data, blood, urine and tissue samples were collected at predefined time points throughout the clinical stay. AKI was defined according to recent recommendations of the Kidney Disease Improving Global Outcome (KDIGO) group. Genetic analysis was performed after patient enrolment was completed.

**Results:**

Between April and December 2014, 150 patients were recruited. The COMT genotypes were distributed as follows: Val/Met 48.7%, Met/Met 29.3%, Val/Val 21.3%. No significant differences were found for demography, comorbidities, or operative strategy according to the underlying COMT genotype. AKI occurred in 35 patients (23.5%) of the total cohort, and no differences were evident between the COMT genotypes (20.5% Met/Met, 24.7% Val/Met, 25.0% Val/Val, *p* = 0.66). There were also no differences in the post-operative period, including ICU or in-hospital stay.

**Conclusions:**

We did not find statistically significant variations in the risk for postoperative AKI, length of ICU or in-hospital stay according to the underlying COMT genotype.

## Background

The Catechol-O-methyltransferase (COMT) represents one of the key enzymes in catecholamine degradation. The common polymorphism rs4680 in exon four is coding for a Val to Met substitution at position 158 and results in increased thermolability of the COMT enzyme: Val/Val being associated with high enzyme activity, Val/Met with intermediate and Met/Met with low enzyme activity [1, 2]. Reduced degradation of catecholamines caused by low COMT enzyme activity leads to persistent vasodilation via, among others, down-regulation and desensitization of α-adrenoceptors and suppression of vasopressin release, thus facilitating prolonged shock with acute kidney injury (AKI) as a consequence [3–5].

Recent studies suggest that this COMT polymorphism is associated with substantial variance in the response to endogenous and exogenous catecholamines, frequently being referred to as “catecholamine resistance” [2, 4]. This phenomenon contributes to hemodynamic responses, e.g. hypotension and might therefore also being related to clinical outcomes. It has been reported that in particular patients undergoing cardiac surgery carrying the COMT Met/Met phenotype are at high risk for vasodilatory shock, AKI and prolonged ICU- and in-hospital stay [4]. However, data are conflicting as the association of COMT genotype with the described outcomes could not be confirmed by recent studies [6, 7].

In the current prospective cohort study, we investigated the prevalence of the various COMT genotypes in patients undergoing cardiac surgery. We also assessed the risk of AKI dependent on COMT genotype as the study’s primary endpoint, assuming higher risk in particular in the COMT low activity phenotype (Met/Met).

## Methods

The study protocol was approved by the Ethics Committee at the University Hospital of Würzburg. All participants provided written informed consent after they were asked at least 24 h prior to surgery (preferably at the day of admission) to participate in the study. Patients (18+ years) were eligible if they were undergoing an elective cardiac surgical procedure involving cardiopulmonary bypass (CPB); such as coronary artery bypass graft (CABG) with or without mammary artery bypass, valve surgery (reconstruction, replacement) with or without removal of the atrial auricle, combined CABG and valve surgery, or surgery of the thoracic aorta. Mild hypothermia (32-34 °C) was applied to all patients during surgery and cardiac arrest resulted from blood cardioplegia (Buckberg). Exclusion criteria included chronic kidney disease greater than stage 3, i.e. eGFR_CKD-EPI_ < 30 ml/min, signs of active infection (clinical assessment), women during pregnancy and lactation and patients on medication with COMT inhibitors, MAO inhibitors or with immunosuppressive therapy.

Collected data comprised routine clinical data on patient characteristics and comorbidities, surgery procedures, anesthesia and ICU details including laboratory data and medication. Information was collected at various predefined time points during the patient’s hospital stay at admission/pre-operatively, at ICU admission, 24 h and 48 h after ICU admission as well as 6 days post-surgery and/or at hospital discharge. Furthermore, standardized interviews were performed by trained study personnel to collect data on medical history, comorbidities and patient related factors e.g. pain at admission and at discharge. Cardiac dysfunction was defined as LVEF< 50% in a standardized echocardiography prior to surgery.

An EDTA-blood sample was drawn preoperatively and COMT genotyping was performed as described previously [8]. In brief, fragments were amplified using the primers COMT-F (5’-TCACCATCGAGATCAACCCC) and COMT-R (5’-ACAACGGGTCAGGCATGCA). Standard polymerase chain reaction (PCR) was carried out in a 25 μl volume containing 60 ng of genomic DNA, 10 pmol of each primer, 200 mM dNTPs, 1 U HotStarTaqTM den, Germany), 50 mM KCl, 1.5 mM MgCl2 DNA polymerase (Qiagen GmbH, Hil- and 10 mM Tris–HCl (pH 8.4). After an initial 15-min denaturation at 94 °C, 35 cycles were carried out consisting of 40 s at 94 °C, 40 s at the annealing temperature of 53 °C and 60 s at 72 °C, followed by a final extension time of 10 min at 72 °C in a Gene Amp PCR System 9700. A fragment length polymorphism (RFLP) assay with the restriction enzyme NlaIII (2 U) was performed as recommended by the manufacturer (New England Biolabs, Frankfurt, Germany) resulting in 65-, 18- and 13-bp bands for the A allele and 83- and 13-bp bands for the G allele, respectively. A total of 8 ml of the digested product were mixed with 12 ml denaturing solution as described above and separated for 3 h on a 15% polyacrylamide gel (acrylamide:bisacrylamide = 49:1; Multigel-Long/Biometra, Gottingen, Germany) containing 1rTBE at 20 V/cm. Bands were visualized by silver-staining. As the genotyping was performed a few weeks after enrollment of all study participants, physicians and study personnel were strictly blinded to COMT genotype.

The study’s primary endpoint was defined as the development of AKI within the first 48 h after surgery according to the most recent recommendations by KDIGO [5] as increase in serum creatinine (SCr) ≥ 0.3 mg/dl within 48 h or increase in SCr ≥ 1.5 times baseline or reduction of urine volume < 0.5 ml/kg/h for at least 6 h. Baseline serum samples were collected during introduction of anesthesia, which is approximately 0.5 h prior to the beginning of surgery and also roughly 1.0 h prior to the start of cardio-pulmonary bypass. Of note, creatinine measurements at hospital admission were checked to exclude preoperative AKI. Subsequently, SCr-measurements were performed as part of clinical routine at postoperative admission on the ICU, 24 h and 48 h post-surgery.

### Statistical methods

Patient demographics are presented as mean and standard deviations, medians and interquartile ranges and number of observations with proportions (%), as appropriate. Differences across COMT-genotype-groups were assessed by ANOVA, Kruskal-Wallis-H-Test and χ^2^-test/Fisher’s exact test, respectively. Uni- and multivariate logistic regression analysis was performed to investigate the risk of AKI dependent on the COMT genotype and clinical as well as surgical parameters. Due to limited statistical power, clinical and surgical factors were time on CPB, as the most important indicator of intraoperative stress [9, 10] and preoperative mortality risk assessed by the EUROSCORE 2 [11]. The score summarizes a variety of patient characteristics including age, gender, chronic pulmonary disease, extracardiac arteriopathy, neurological dysfunction disease, previous cardiac surgery, SCr, active endocarditis (which represented an exclusion criterion for the current study) and critical preoperative state. Two-sided *p*-values of ≤0.05 were considered as statistically significant. Statistical analyses were performed using SPSS Versions 22 and 23.

### Power calculation

The current study was a pilot study to investigate the hypothesis that the various COMT genotypes can be observed according to the Hardy-Weinberg equilibrium and that the resulting phenotypes would carry different risk for AKI. The power calculation was based on the following assumptions: the prevalence of the COMT polymorphism would be Val/Val (25%), Val/Met (50%) and Met/Met (25%). The distribution of AKI across COMT phenotype has been described as Met/Met: 31%, Val/Met: 19.5%, and Val/Val: 13.5%, respectively [4]. Since we observed a higher incidence of AKI after cardiac surgery in a previous independent study at our division, i.e. about 60% [12], we assumed AKI risk in the Val/Val group of the current study to be 20%. For the purpose of being a pilot study to confirm the role of the COMT polymorphism in cardiac surgery, we planned to enroll a total number of *n* = 150 patients, resulting in an assumed distribution of COMT genotype of Val/Val *n* = 37, Val/Met *n* = 74, Met/Met n = 37, total *n* = 148, drop out *n* = 2. This approach allowed for rejecting the null-hypothesis that the risk for AKI does not vary across phenotypes (H_0_: AKI_Val/Val_ = AKI_Val/Met_ = AKI_Met/Met_) with a power of 0.80 and alpha of ≤0.05, if the incidence in the remaining groups are AKI-risk Val/Met = 29% and AKI-risk Met/Met = 53%. Of note, it was planned by protocol, that if signals can be observed that supported our hypothesis that the Met/Met (or any other) genotype was associated with AKI, the sample size shall be increased and to focus on modifiable surgery-related factors as the basis for interventional studies.

## Results

Eligible patients were screened for participation between April and December 2014. During the study, 165 patients (of 915 per se eligible subjects within this time-period undergoing surgery at our department) were included matching our in- and exclusion criteria. There were 15 dropouts due to unplanned off-pump surgery, withdrawal of consent or missing genotypes; thus 150 patients were included in the final analysis.

Patient characteristics of the entire cohort and stratified according to COMT genotype are displayed in Table 1. Patients were on average 67 years old (median) and 28.2% were female. Eighty-four patients (55.6%) patients had at least one CABG and 80 (53.0%) received valve surgery. Median operation duration was 215 min, and median CPB duration was 105 min. Preoperative kidney function was described by median SCr 0.93 mg/dl, reflecting an eGFR of 81.1 ml/min/1.73m^2^. Median EUROSCORE 2 was 1.7. Seventy-four (49%) patients had cardiac dysfunction, i.e. left ventricular ejection fraction (LV-EF) < 50%. The prevalence of cardiac dysfunction was lowest in the Val/Met group (31.5%), while in the Val/Val group the prevalence was 71.9%. There were no other significant differences regarding demography, medical history and surgical approach between the three COMT genotypes.

**Table 1:** Patient characteristics and surgery data

Variable	All patients (*n* = 150)	Met/Met (*n* = 44)	Val/Met (*n* = 73)	Val/Val (*n* = 32)	*p*-value
Age (yrs)	67 (60 – 75)	69 (62 – 76)	67 (57 – 74)	65 (61 – 76)	0.41
Female n (%)	42 (28.2)	9 (20.5)	21 (28.8)	12 (37.5)	0.28
Weight (kg)	82.0 (73.0 – 92.0)	81.5 (76.5 – 92.5)	82.0 (74.0 – 92.0)	78.5 (69.5 – 90.0)	0.46
Height (cm)	172.5 (165.0 – 179.0)	175.0 (170.0 – 178.0)	172.0 (167.0 – 179.0)	171.0 (162.5 – 179.5)	0.39
BMI (kg/m^2^)	27.8 (±4.4)	27.9 (±4.0)	27.8 (±4.9)	27.5 (±4.1)	0.89
Cardiac Dysfunction n (%)	69 (46.3)	23 (52.3)	23 (31.5)	23 (71.9)	< 0.001
Pulm. Hypertension n (%)	10 (6.6)	2 (5.7)	5 (8.2)	3 (12.0)	0.69
FEV1 (l/s)	2.64 (1.92 – 3.15)	2.83 (2.31 – 3.15)	2.64 (2.04 – 3.15)	2.50 (1.79 – 3.16)	0.67
AF n (%)	16 (10.6)	6 (13.6)	8 (11.0)	1 (3.1)	0.31
PM n (%)	7 (4.6)	2 (4.5)	4 (5.5)	1 (3.1)	1.00
History of Stroke/TIA n (%)	48 (32.2)	18 (40.9)	22 (30.1)	8 (25.0)	0.32
Crea, pre-OP (mg/dl)	0.93 (0.77 – 1.09)	0.96 (0.76 – 1.11)	0.91 (0.77 – 1.08)	0.93 (0.76 – 1.07)	0.76
eGFR, pre-OP (ml/min)	81.1 (63.6 – 95.1)	79.7 (63.0 – 94.9)	84.6 (67.7 – 95.8)	82.3 (66.5 – 94.1)	0.74
Euroscore II (%)	1.7 (1 – 2.7)	1.8 (1.0 – 3.1)	1.6 (1.0 – 2.7)	1.6 (1.0 – 3.1)	0.75
Surgery					
CABG n (%)	84 (55.6)	25 (56.8)	36 (50.7)	23 (71.9)	0.13
Valve n (%)	80 (53.0)	24 (55.8)	39 (56.5)	16 (51.6)	0.88
Aortic surgery n %)	15 (9.9)	3 (7.0)	9 (12.9)	2 (6.3)	0.57
OP Duration (min)	215.0 (185.5 – 252.5)	202 (182.5 – 237.0)	218 (189.0 – 260.0)	224 (181 – 253)	0.39
CPB Duration (min)	105 (80.5 – 127.5)	93.5 (78.25 – 121.25)	112.5 (85.0 – 135.0)	108.0 (75.5 – 120.5)	0.19
X-Clamp n (%)	143 (94.7)	42 (95.5)	70 (97.2)	30 (93.8)	0.65
Circulatory arrest n (%)	11 (7.3)	2 (5.4)	7 (11.3)	1 (3.8)	0.50
Complication n (%)	11 (7.3)	4 (9.1)	6 (8.5)	1 (3.1)	0.60

Data are mean (±SD), number (%) or median (IQR); p-values were taken from univariate analyses. *BMI* Body Mass Index; Cardiac Dysfunction: LV-EF < 50% or Diastolic Dysfunction I°, Pulm. Hypertension: Pulmonary Hypertension, *FEV1* Forced Expiratory Volume within 1 s, *AF* Atrial Fibrillation, *PM* Pacemaker, *TIA* Transiant Ischaemic Attack, *Crea* creatinine, *GFR* Glomerular Filtration Rate, *CABG* Coronary Artery Bypass Grafting, *Valve* Aortic-, Mitral- or Triskuspidal Valve surgery, *OP* Operation, *CPB* Cardio-pulmonary Bypass, *X-Clamp* Aortic Cross Clamping; Complication: Severe Bleeding, Rethoracotomy, Mortality

### Postoperative incidence of AKI

A total of 35 patients (23.5%) experienced AKI within 48 h after surgery (Table 2). There was no difference between the three COMT genotypes regarding the incidence of AKI within 48 h. Neither median values of urinary output over 24 h at post-OP day 1 and 2 nor median SCr-levels nor the incidence of specific stages of AKI indicated statistically significant differences between the three genotypes (Table 2). Two patients, both in the COMT Val/Met group, required renal replacement therapy during their ICU stay. The COMT genotype was not associated with the incidence of AKI in univariate and multivariate logistic regression analysis. An increased risk of AKI was found for patients with longer CPB-duration (*p* = 0.02), and a statistically non-significant trend was observed for patients with higher EUROSCORE 2 values (*p* = 0.09). In multivariable analysis, only time on CPB remained statistically significant (*p* = 0.049) in the model (data not shown).

**Table 2:** Clinical outcomes

Variable	All patients	Met/Met (*n* = 44)	Val/Met (*n* = 73)	Val/Val (*n* = 32)	*p*-value
Clinical events/parameters within 48 h after surgery					
AKI within 48 h, n (%) (primary endpoint)	35 (23.5)	9 (20.5)	18 (24.7)	8 (25.0)	0.85
Stage I	27 (18.1)	8 (18.2)	13 (17.8)	6 (18.8)	0.99
Stage II	6 (4.0)	1 (2.3)	3 (4.1)	2 (6.3)	0.69
Stage III	2 (1.3)	0	2 (2.7)	0	0.35
SCr _ICU admission_ (mg/dl)	1.01 (0.85 – 1.21)	1.00 (0.85 – 1.21)	0.98 (0.82 – 1.22)	1.07 (0.94 – 1.17)	0.85
SCr_24h post-OP_ (mg/dl)	0.99 (0.82 – 1.22)	1.04 (0.82 – 1.23)	0.97 (0.81 – 1.23)	0.90 (1.00 – 1.20)	0.69
SCr _48h post-OP_ (mg/dl)	0.99 (0.83 – 1.27)	1.00 (0.81 – 1.29)	0.95 (0.83 – 1.38)	1.02 (0.88 – 1.18)	0.96
Urinary output, day of surgery (ml/24 h)	2350 (1820 – 2960)	2350 (1900 – 2940)	2370 (1860 – 2860)	2150 (1650 – 3070)	0.84
Urinary output, post-OP day 1 (ml/24 h)	2650 (2300 – 3053)	2550 (2250 – 2950)	2850 (2350 – 3195)	2500 (2275 – 2825)	0.11
Clinical events during hospital stay					
AKI during hospital stay, n (%)	45 (30.2)	11 (25.0)	24 (32.9)	10 (31.3)	0.66
Crea, discharge (mg/dl)	1.09 (0.87 – 1.27)	1.10 (0.94 – 1.27)	1.04 (0.84 – 1.28)	1.11 (0.87 – 1.22)	0.88
eGFR, discharge (mg/dl)	68.7 (54.0 – 83.5)	64.5 (54.6 – 77.0)	68.8 (52.6 – 89.4)	68.2 (56.4 – 81.5)	0.79
ICU stay (h)	24.87 (22.13 – 47.77)	24.9 (22.3 – 69.9)	24.2 (22.1 – 46.2)	27.3 (22.4 – 47.1)	0.80
Hospital stay (d)	7 (6 – 8)	7 (6 – 8)	7 (6 – 8)	6 (6 – 8)	0.79
Mechanical ventilation (h)	12.42 (10.65 – 16.38)	12.3 (9.3 – 15.4)	12.3 (11.0 – 16.4)	14.1 (11.0 – 17.3)	0.29

Data are mean (±SD), number (%) or median (IQR); p-values were taken from univariate analyses. *AKI* acute kidney injury, *SCr* Serum Creatinine, *Crea* Creatinine, *GFR* Glomerular Filtration Rate, *ICU* Intensive Care Unit

To further understand the role of cardiac dysfunction, we performed additional analyses but did not find any meaningful and significant association of the COMT genotype, cardiac dysfunction and the occurrence of postoperative AKI. Also in logistic regression analyses, cardiac dysfunction was not associated with the incidence of AKI neither in univariate (OR 0.7, 95% CI 0.33 – 1.51, *p* = 0.4), nor in multivariate modelling (OR 0.70, 95% CI 0.31 – 1.57, *p* = 0.4) controlling for COMT genotype and also including the interaction of COMT genotype and cardiac dysfunction.

## Discussion

In our prospective cohort study, we found that the patients undergoing cardiac surgery reflected the described distribution of the three COMT genotypes according to the Hardy-Weinberg Equilibrium. However, we could not detect any statistically significant risk-variation of postoperative AKI dependent on COMT genotype. Furthermore, no statistically significant differences in pre-operative parameters, operative strategy and other post-operative outcomes such as time on mechanical ventilation, length of ICU stay or hospital stay were observed.

The COMT genotype within our study population was distributed as previously described with proportions according to the Hardy Weinberg Equilibrium [2, 4]. However, patients carrying either genotype did not differ in demographic parameters, comorbid conditions and medical history. It seems plausible that variations in catecholamine degradation based on COMT genotype may be a result of linked polymorphisms, e.g. those with the Val/Val-genotype could present with more severe CVD and accelerated CVD-progression only if they additionally have altered mRNA secondary structure [13], which has been demonstrated in a Japanese population [14]. Furthermore, although the specific COMT genotype was not known by the treating physicians, the pre-operative clinical phenotype and also intra-operative situations based on varying catecholamine-resistance due to the COMT polymorphism, may have led to differences in the chosen operative and narcotic strategy, intra operative and early postoperative complications. We could not detect any association of the COMT genotype with these factors, and in multivariate modelling only the time on CPB was associated with a higher risk for post-operative AKI. It may be only due to limited statistical power, that the association of preoperative status according to EUROSCORE 2 with a higher risk for AKI was only observed as a non-significant trend. We hypothesize that this relationship whould have become statistically significant if more subjects had been enrolled.

The incidence of AKI was lower than in previous studies at our centre [12, 15] which might also be explained by the fact that we excluded patients with advanced stages of CKD (i.e., eGFR < 30 ml/min/1.73m^2^). These patients per se are at increased risk for AKI [16]. On the other hand, standard fluid management during surgery has been modified in the last years including our Division, and colloidal infusions have been avoided in exchange to crystalline solutions [17]. This certainly might have an impact on organ perfusion, severity and duration of shock and thus on kidney function during surgery and on the ICU [18]. However, the overall incidence of AKI in the current study overall met our assumptions applied to the power analysis: the incidence of AKI in the Val/Val and Val/Met groups, respectively, were as high as expected. Yet, in the Met/Met group which was reported to carry higher risk for AKI, we could not observe a higher incidence of AKI, certainly not as being of 53% according to our assumptions. In fact, we found 20.5% of the patients with AKI in this particular COMT group. Therefore, as our data did not support our preset hypothesis, we followed our protocol and refrained from enrolling more patients.

It is a major strength of our study that baseline SCr was prospectively measured at a defined time-point (during introduction of anesthesia) followed by subsequent measurements in clinical routine up to 48 h on the ICU. This approach excluded pre-operative AKI and also allowed for classification of AKI following the most recent KDIGO guidelines. We could not observe any variation in the risk of AKI according to COMT genotype, neither in slight elevations of SCr (i.e. AKI stage 1), nor in higher stages of AKI. Only two patients required hemodialysis treatment for AKI and both were in the (theoretically) intermediate-risk COMT Val/Met group.

Evidence is robust that adverse peri- and postoperative events, including vasodilatory or cardiogenic shock and AKI are related to worse subsequent outcomes; such as the development of chronic kidney disease (CKD), and also of systolic and/or diastolic left ventricular dysfunction, congestive heart failure (CHF) and mortality [6, 7, 19]. Particularly AKI has been shown to be related to short- and long term mortality and morbidity in critical care patients [6, 7, 19] and also to the development and/or progression of CKD, cardiac dysfunction and overt heart failure [20–25]. Impairments in kidney function and in particular those patients in whom kidney parameters numerically recover to normal values were underestimated in the past and even slight changes in kidney function and have been identified as a major complication with significant prognostic implications [22, 23, 25–27].

Data on the risk of AKI being dependent on the COMT genotype is conflicting. In particular, two recent publications report on associations between COMT and AKI or renal damage, respectively. Herein, the Met/Met phenotype of the COMT enzyme was associated with an increased risk of AKI [4]. This was explained be a decreased catecholamine degradation in association with shock caused by the underlying COMT polymorphism [4]. In a more recent attempt the same group could not confirm their previous finding in an independent cohort [7]. Instead, they describe an elevation of renal stress markers in relation to the COMT genotype, however, without consecutive AKI. Moreover, in a retrospective approach, another group was also not able to not show an association between COMT genotype and RIFLE-AKI after cardiac surgery [4, 6, 7].

While all papers used the RIFLE classification for AKI definition, we used the latest definition by the Kidney Disease Improving Global Outcome (KDIGO) published in 2012 [4–7]. The authors stated that the RIFLE classification might be more robust for AKI diagnosis after cardiac surgery, but most recent evidence suggests that the KDIGO definition with its strict increase of SCr of 0.3 ml/dl within 48 h definition has an impact on short- and longterm outcome [20, 21, 28–32]. Even slight changes and rises in SCr levels are associated with a significant increase of early postoperative mortality [29]. Mild AKI are associated with enhanced mortality and rehospitalisation rates after cardiac surgery also [20, 28].

The disparities between the RIFLE and the KDIGO definition of AKI might result in a different incidence of AKI; in contrast to the KDIGO definition, particularly mild alterations in kidney function are not being classified as AKI according to RIFLE. Therefore, any association of the COMT genotype with AKI according to RIFLE may be detected predominantly in more severe stages of AKI. Most of the AKI episodes in our study were of stage 1 and the number of more severe AKI stages was very limited. Therefore the statistical power of our study may be too small to observe significant differences in the AKI incidence according to COMT genotype. Yet, as we did not find any signal for any association of COMT genotype with the risk of AKI, we believe that there may not exist any meaningful association.

In our analyses, we found an association between cardiac dysfunction and the COMT polymorphism, which has not been described in the literature. We found a higher prevalence of cardiac dysfunction in the Val/Val genotype group, while the Val/Met genotype was associated with a low prevalence of impaired LV function. Further analyses did not reveal any association between cardiac dysfunction and the incidence of AKI. Our findings of a potentially existing clinical relation of cardiac dysfunction an the underlying COMT genotype, admittedly being based on limited statistical power, need to be confirmed by future studies.

We are aware of several limitations of our study. First, reflecting a pilot study, the sample size is limited to detect small differences between the COMT phenotypes and postoperative outcomes and also to control for covariates in multivariable modelling. Overfitting the multivariate models might also be possible and therefore missing or even finding any associations by chance is truly possible. Second, there is no standardized operating procedure at our department considering fluid or catecholamine management of the patients, therefore treatment bias cannot be excluded. However, the treating physicians and nurses as well as the study personnel were unware of the underlying COMT polymorphism, as the COMT genotype were measured after completion of enrolment. Thirds, during 9 months study duration, a total of 915 patients would have been eligible, but due to limited resources on study personnel, only 165 patients were enrolled. Therefore, selection bias is conceivable, but we assume that our study sample overall reflects most of all eligible patients at our center: the study population was distributed by COMT genotype as expected following the Hardy-Weinberg-equilibrium with no differences in pre-operative patient characteristics. It is thus unlikely that a meaningful proportion of Met/Met patients which are theoretically at very high risk for AKI were not enrolled due to whatever clinical reasons, but definitely unconscious to the COMT genotype.

## Conclusions

The COMT rs4680 genotype was not associated with statistically significant differences in preoperative phenotypes, the surgical strategy, intraoperative and early postoperative complications. We did not find variations of the risk for postoperative AKI according to COMT genotype.

## Abbreviations

AKI: Acute kidney injury
CABG: Coronary artery bypass graft
CKD: Chronic kidney disease
COMT: Catechol-O-methyltransferase
CPB: Cardio pulmonary bypass
CVD: Cardio vascular disease
ICU: Intensive Care Unit
KDIGO: Kidney Disease Improving Global Outcome
LV: Left ventricle
LV-EF: Left ventricular ejection fraction
RIFLE: Risk Injury Failure Loss End-Stage Kidney Disease
SCr: Serum creatinine
